# A Novel Tongue Coating Segmentation Method Based on Improved TransUNet

**DOI:** 10.3390/s24144455

**Published:** 2024-07-10

**Authors:** Jiaze Wu, Zijian Li, Yiheng Cai, Hao Liang, Long Zhou, Ming Chen, Jing Guan

**Affiliations:** 1 School of Traditional Chinese Medicine, Beijing University of Chinese Medicine, Beijing 102488, China; 20220931017@bucm.edu.cn (J.W.); lizijian202209@163.com (Z.L.); zhoulong_bucm@163.com (L.Z.); 2 Department of Information, Beijing University of Technology, Beijing 100124, China; caiyiheng@bjut.edu.cn; 3 School of Chinese Medicine, Hunan University of Chinese Medicine, Changsha 410208, China; lianghao@hnucm.edu.cn

**Keywords:** computer vision, deep learning, medical image segmentation, tongue diagnosis, TransUNet

## Abstract

Background: As an important part of the tongue, the tongue coating is closely associated with different disorders and has major diagnostic benefits. This study aims to construct a neural network model that can perform complex tongue coating segmentation. This addresses the issue of tongue coating segmentation in intelligent tongue diagnosis automation. Method: This work proposes an improved TransUNet to segment the tongue coating. We introduced a transformer as a self-attention mechanism to capture the semantic information in the high-level features of the encoder. At the same time, the subtraction feature pyramid (SFP) and visual regional enhancer (VRE) were constructed to minimize the redundant information transmitted by skip connections and improve the spatial detail information in the low-level features of the encoder. Results: Comparative and ablation experimental findings indicate that our model has an accuracy of 96.36%, a precision of 96.26%, a dice of 96.76%, a recall of 97.43%, and an IoU of 93.81%. Unlike the reference model, our model achieves the best segmentation effect. Conclusion: The improved TransUNet proposed here can achieve precise segmentation of complex tongue images. This provides an effective technique for the automatic extraction in images of the tongue coating, contributing to the automation and accuracy of tongue diagnosis.

## 1. Introduction

Tongue diagnosis is a major direct and objective basis for clinical diagnosis and treatment in traditional Chinese medicine. It plays an important role in disease diagnosis owing to its noninvasive and convenient features [1]. Typical normal tongue appearance is characterized by a moderate tongue shape, light-red tongue body, and thin white tongue coating; abnormal tongue appearance is considered an early warning signal of potential health problems [2]. During tongue diagnosis, the tongue body and tongue coating are two vital objects to observe since they provide essential information regarding the health status of the patient. Unlike the tongue body, the tongue coating has significant benefits in some aspects of disease diagnosis. For example, it can quickly indicate the condition of the digestive system [3]. In addition, its color and texture are prone to change and can be observed readily, allowing physicians to monitor the progression of a condition and of treatment efficacy by regularly observing changes. In recent years, with the introduction of novel research methods, several scholars have made some strides in the field of tongue coating research. Their studies have enriched our understanding of the tongue coating and further corroborated the strong link that exists between the tongue coating and diseases. For example, Jiahui Chen et al. [4] used the pressure cycling technique and data-independent acquisition (PCT-DIA) mass spectrometry to extract and identify proteins from the tongue samples of 180 gastric cancer patients and 185 nongastric cancer patients. They investigated the temporal stability of the tongue-coated proteins through a time-series cohort study and finally constructed a gastric cancer screening model based on microbial-derived tongue-coated proteins. Marwan Mansoor Ali Mohammed et al. [5] carried out a systematic analysis of studies comparing the tongue coating microbial communities of cancerous or precancerous cases and healthy controls based on NGS technology in multiple databases such as PubMed and Web of Science. Their findings found a relationship between changes in tongue coating microbial communities and various diseases, specifically cancer. Yuren Zhang et al. [6] utilized 16S rRNA gene sequencing to analyze tongue coating samples from 60 patients with primary liver cancer and 25 healthy controls and revealed specific clinical features as well as bacterial structures in primary liver cancer patients with differences in tongue coatings. In conclusion, as a vital component of the tongue, the tongue coating helps physicians to better understand the onset and progression of disease as well as provides key information on disease prognosis.

With the continuous development of smart healthcare, smart tongue diagnosis has made remarkable strides, due in major part to the strong support provided by tongue segmentation techniques [7,8,9]. Current state-of-the-art methods primarily focus on tongue body segmentation and rarely involve tongue coating segmentation. Nonetheless, as mentioned above, the many benefits of the tongue coating in diagnosis make it a key object for physicians to understand the occurrence, development, and prognosis of diseases. Thus, segmenting the tongue coating from the tongue body is important for intelligent tongue diagnosis. Xu Wang et al. [10] constructed GreasyCoatNet to identify a greasy tongue coating and applied it to the diagnosis of COVID-19. However, the authors mentioned that background information outside the tongue coating may affect the performance of the model. They used Colabeler software (version 2.0.4; Hangzhou Kuaiyi Technology Co., Ltd., Hangzhou, China) to manually mark this, but accuracy and efficiency cannot be guaranteed. Shixuan Dai et al. investigated the application of intelligent tongue image analysis in conjunction with microbiomes in the diagnosis of MAFLD. This process analyzes the texture and color indices of the tongue coating segmented from the tongue body and studies their relationship with the microbiota of the tongue coating. Jun Li et al. [11] and Tao Jiang et al. [12] studied the relationship between the tongue coating and chronic diseases such as diabetes, hypertension, and hyperlipidemia by building diagnostic models. Therefore, if the tongue coating can be quickly and accurately segmented from the tongue body, excluding the influence of irrelevant factors, it will be of great benefit to intelligent tongue diagnosis. Existing methods for tongue segmentation fall into two major categories, i.e., feature engineering and deep learning. Feature engineering is based on algorithms including thresholds, edges, and regions and uses a priori knowledge for segmentation [13,14,15]. This approach has the benefit of having a simple model, quick training, and a dataset that does not require expert labeling. However, it requires higher-quality images, and issues with low segmentation accuracy and poor robustness must be solved. Deep learning has achieved significant success in the field of medical image segmentation by creating neural network models that automatically recognize tongue features and carry out segmentation [16,17,18]. The neural network model exhibits robustness and segmentation accuracy for various tongue images despite its relative complexity, lengthy training period, and requirement of expert labeling of the dataset. Unlike tongue body segmentation, tongue coating segmentation faces greater challenges. Due to the similarity in color of and unclear boundaries between the tongue coating and tongue body, it is challenging for feature engineering to achieve high accuracy and robustness in tongue coating segmentation. Therefore, the use of deep learning techniques to construct neural network models and automatically recognize and extract tongue features for segmentation is anticipated to solve this complex challenge. U-Net, proposed by Ronneberger et al. [19], is popular in the field of medical image segmentation; it is characterized by the clever use of encoding-layer features in the decoding process, achieving a perfect combination of being lightweight and having high performance, and it occupies a dominant position in the field of medical image segmentation. With the success of the transformer in numerous natural language processing tasks, Alexey Dosovitskiy et al. [20] introduced it into the field of image processing and proposed a vision transformer in an attempt to investigate the potential use and applicability of the transformer’s self-attention mechanism, parallelization capacity, and long-range dependency capture benefits in the field of image processing [21]. TransUNet, proposed by Jiening Chen et al. [22], combines the benefits of UNet and vision transformer. It treats high-level features of the encoder as sequence inputs to the transformer to handle long-distance semantic relationships before combining them with low-level features of the encoder through a U-shaped structure in the decoder via skip connections to make up for missing spatial detail information.

Our objective is to automatically segment the tongue coating from a complex tongue body, independent of the diversity of its appearance and boundary ambiguity, without human intervention. TransUNet can efficiently capture the long-distance dependencies between tongue semantics, but at the same time, it pays insufficient attention to the spatial detail information of the tongue coating edges, yielding inaccurate tongue segmentation results. Therefore, we propose an improved TransUNet that preserves and improves the spatial detail information of tongue coating edges under the premise of guaranteeing the long-distance dependencies between tongue semantics, thereby improving the accuracy of tongue coating segmentation. The model has been trained and tested on a dataset containing 300 sets of tongue images. The comparison and ablation experiments indicate that our model outperforms UNet, UNet++, and SegNet in tongue coating segmentation. Furthermore, it improves the regional detail information of the encoder’s low-level features, unlike TransUNet, which improves the final tongue coating segmentation accuracy.

Overall, our contributions can be summarized as follows:

1. We innovatively introduce TransUNet into the task of tongue coating segmentation, integrating semantic information from the high-level features captured by the transformer and spatial information from the low-level features of the encoder through the skip connection structure of UNet. This achieves the complete and continuous segmentation of the tongue coating from the tongue body, aiming to solve the problem of tongue coating segmentation in intelligent tongue diagnosis.
2. We improve and design the subtraction feature pyramid (SFP) and visual regional enhancer (VRE) modules. SFP is used to reduce redundant information in low-level encoder features and focus on local spatial details; VRE is used to enrich spatial detail information in low-level features, reduce significant differences between high-level and low-level features, and enable more effective fusion.
3. Comparative experiments and ablation experiments show that our model has superior overall performance compared to the commonly used UNet, UNet++, and SegNet models for medical image segmentation on the same dataset. Furthermore, it can also better cope with irregular tongue coatings such as tooth marks, cracks, peeling, etc., with unclear boundaries, irregular shapes, and irregular distribution.

## 2. Materials and Methods

**Figure 1:**
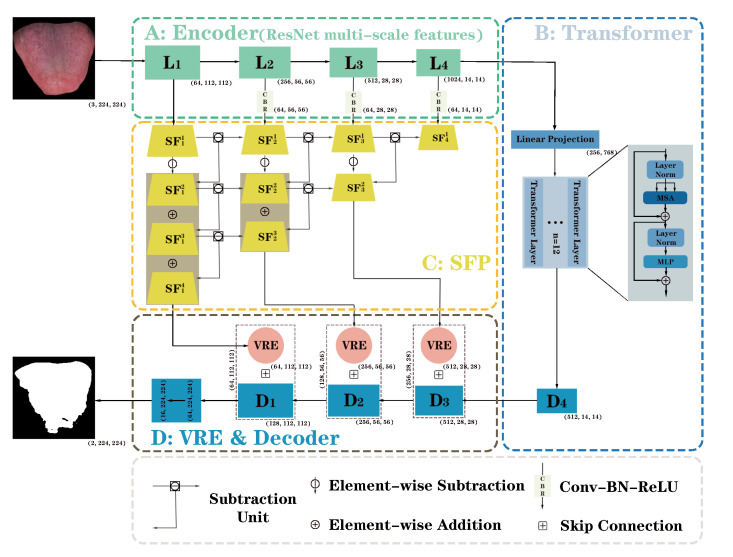
Model structure.

Our model (Figure 1) involves three stages, i.e., a feature extraction stage (Figure 1A,B), a feature fusion stage (Figure 1C,D), and a prediction stage. Specifically, in the feature extraction stage, ResNet-50 [23] is used to extract multiscale features of the tongue image and feed high-level features into the transformer to further extract the long-distance dependencies between semantics. ResNet-50 benefits from the excellent learning ability and efficiency brought by residual structures, the widespread availability of pretrained models, and the excellent generalization performance demonstrated in various tasks, making it the preferred feature extraction model for balancing performance and resource requirements. In the feature fusion stage, we designed the subtraction feature pyramid (SFP) and visual regional enhancer (VRE) to improve the spatial detail information in the low-level features and reduce redundant information conveyed by skip connections. Subsequently, the low-level features processed by SFP and VRE and high-level features processed by the transformer undergo feature fusion in the decoder before gradually being restored to the original resolution by deconvolution. In the prediction stage, we test the model using a combined loss function comprising CE loss, dice loss, and focal loss, considering the category imbalance and the difficulty of feature learning.

Generally, features at different levels in the encoder have different properties in a U-shaped structure. The high-level features have a strong capacity to characterize semantic information; however, the resolution is low and the ability to characterize spatial information is insufficient. On the other hand, the low-level features exhibit a strong capacity to characterize spatial information and a high resolution; however, the ability to characterize semantic information is weak. Therefore, the combined use of different levels of features is conducive to improving model stability and reliability. Of note, scale plays a key role in capturing the contextual information of these features; multiscale features can cause better model performance [24]. Current multiscale-based strategies can be broadly categorized into two types, i.e., interlayer multiscale structures and intralayer multiscale structures. The former is based on an encoder extracting features at different scales and progressively fusing them in a decoder, e.g., U-Net and its variants [19,25,26,27]. The latter is usually equipped with a multiscale module, e.g., ASPP and its variants [28,29,30] by building parallel multibranch convolutional layers with different expansion rates for a rich combination of receptive fields. Nonetheless, for interlayer multiscale structures, most of the existing methods directly use element summing or splicing to fuse any two levels of features of the encoder and transmit them to the decoder via skip connections. These operations do not pay much attention to the different information between the features of different levels, which generates redundant information and weakens the level-specific features. This yields a model that is unable to balance accurate localization and fine boundary refinement, specifically the spatial information in the low-level features. Therefore, inspired by Xiaoqi Zhao et al. [31] and Yu Quan et al. [32], we introduce both inter- and intralayer multiscale structures to construct SFP and VRE for the model to balance the precise localization and fine boundary refinement in order to effectively utilize the features of different levels and improve spatial detail information in the low-level features.

### 2.1. Subtraction Feature Pyramid

The structure of the SFP is shown in Figure 1C. The objective is to build an interlayer multiscale structure that minimizes redundant information in the low-level features of the encoder and better uses encoder-specific level features. We define a subtraction unit (1), $L_{A}$ and $L_{B}$ for features of ResNet at different scales, ⊝ for the element-wise subtraction operation, |·| for computing the absolute value, and CBR(·) as the module that unites the 3 × 3 convolution, BN, and ReLU. The number of channels is initially reduced to 64 using CBR for each level of encoder features, respectively, to reduce the parameter quantity for subsequent operations; subsequently, the features of each level are inputted into the SFP, and the level difference is obtained after calculating the features of neighboring levels many times level by level using the subtraction unit. The encoder feature subtracts the sum of level differences and then multiplies them by a learnable weight parameter to obtain the feature at each level. In the multiscale in multiscale subtraction module (MMSM) designed by Xiaoqi Zhao et al. [31], the encoder feature performs a summation operation on level differences in order to make the low-level features incorporate semantic information of the high-level features, whereas our model adopts the transformer to specifically deal with the semantic information of the high-level features. Thus, the encoder feature conducts a subtraction operation on the level difference sum to enable low-level features to focus on local spatial detail information.

(1) $$SU=CBR(\left|L_{A}⊖L_{B}\right|)$$

### 2.2. Visual Regional Enhancer

The U-shaped structure combines convolutional coding features and deconvolutional decoding features at the same stage via skip connections. The encoder features are some low-level features, whereas the decoder features are high-level features after multiple convolutional operations and transformer processing. Significant differences exist between high-level and low-level features, which may result in incompatibility [26], and direct fusion may adversely influence the results. Therefore, to minimize the adverse effects and guarantee the accuracy and reliability of the segmentation outcomes, it is necessary to enrich the spatial detail information in the low-level features. Yu Quan et al. [32] resolved this issue by designing the learnable visual center (LVC) module for aggregating local spatial features. Briefly, LVC transforms the input features shaped like C×H×W into a set of C-dimensional features $X=\left\{{\overset{ˇ}{x}}_{1},{\overset{ˇ}{x}}_{2},\cdots ,{\overset{ˇ}{x}}_{N}\right\}$, where N=H×W is the total number of features. Thereafter, X is inputted into the codebook to compute the weight coefficients *w* (2). The codebook comprises two sets of learnable parameters: 1. codewords: $B=\left\{b_{1},b_{2},\cdots ,b_{K}\right\}$; 2. factors: $S=\left\{s_{1},s_{2},\cdots ,s_{K}\right\}$. Eventually, *X* is multiplied with w to obtain the features improved with spatial detail information.

(2) $$w=Sigmoid(Linear\left(\sum_{k=1}^{K}BRM\left(\sum_{i=1}^{N}\frac{exp^{-s_{k}{∥{\overset{ˇ}{x}}_{i}-b_{k}∥}^{2}}}{\sum_{j=1}^{K}exp^{-s_{j}{∥{\overset{ˇ}{x}}_{i}-b_{j}∥}^{2}}}\left({\overset{ˇ}{x}}_{i}-b_{k}\right)\right)\right))$$

Specifically, the codebook uses factor S to continuously map ${\overset{ˇ}{x}}_{i}$ and $b_{k}$ to corresponding information. ${\overset{ˇ}{x}}_{i}-b_{k}$ is the difference between *N* input features *X* and *K* codewords *B*, and ∥·∥ is the L2 parameter operation. $\frac{e^{-s_{k}{∥{\overset{ˇ}{x}}_{i}-b_{k}∥}^{2}}}{\sum_{j=1}^{K}e^{-s_{j}{∥{\overset{ˇ}{x}}_{i}-b_{j}∥}^{2}}}$ is the computed learnable weight, $s_{k}$ is the factor of the *k*-th codeword, and $s_{k}{∥{\overset{ˇ}{x}}_{i}-b_{k}∥}^{2}$ is the output of the *k*-th codeword. The learnable weight is multiplied by ${\overset{ˇ}{x}}_{i}-b_{k}$ to obtain information about pixel *i* relative to codeword *k*. The *N* results are summed and averagely fused via BRM (BN-ReLU-Mean) to obtain the entire channel (C) value relative to the *k*-th codeword. For the information from the image, the complete information about the *K* codewords *B* of the image *X* can be obtained by adding the *K* results. Afterward, linear converts the results to size C×1×1 and obtains the weight coefficients *w* by Sigmoid.

**Figure 2:**
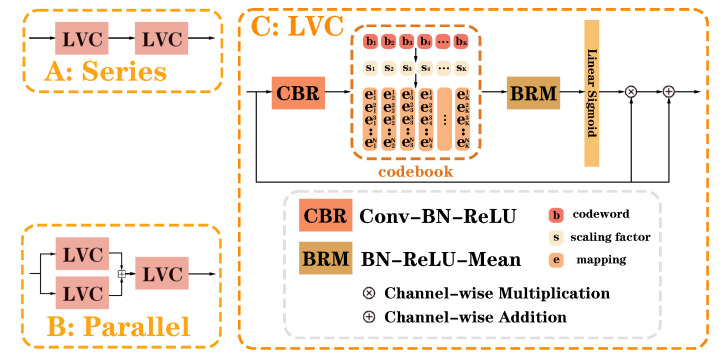
VRE structure.

Based on LVC, we designed VRE (Figure 2A,B), which is an intralayer multiscale structure with LVC as the coding unit. We attempted to connect LVC in series and parallel, respectively, and the experimental results revealed that the series structure has a better effect.

### 2.3. Loss

The loss function of our model comprises three parts: CELoss, DiceLoss, and FocalLoss. CELoss can be used in most image segmentation scenarios. It evaluates the class prediction of each pixel and then averages all pixels; thus, all pixels can be considered equally recognizable. However, class imbalance often occurs in medical image segmentation. This will cause training to be dominated by classes with more pixels, making it difficult to recognize features of smaller objects, thus reducing the efficacy of the model. In terms of tongue appearance, it can be divided into two categories: tongue coating and background. In the tongue coating segmentation, the tongue coating occupies a larger part of the tongue image. If CELoss is used, it becomes difficult to correctly classify the tongue coating edges and isolated small pieces of tongue coating. Therefore, we introduced region-dependent dice loss [33], which tests the class prediction of each pixel and its neighboring pixels to alleviate the negative effect of class imbalance. Additionally, regarding the difficulty in recognizing the fuzzy boundary between the tongue coating and tongue body, we introduced FocalLoss [34], which adds weight to the loss value based on the difficulty of category learning, i.e., adding smaller weights to categories that are easy to learn (background) and adding larger weights to categories that are difficult to recognize (tongue coating). In summary, we constructed a comprehensive loss function (3) where $w_{1}$, $w_{2}$, and $w_{3}$ are learnable weight parameters, with initial values of 0.3, 0.4, and 0.3, respectively. Generally speaking, when the loss of a model is the weighted sum of multiple loss functions, the weights of different loss functions are manually set based on their importance, and the weighted values either are uniform or require manual fine-tuning. However, manually fine-tuning these weighted values to achieve optimal results is expensive and difficult. Therefore, we designed a loss module consisting of CELoss, DiceLoss, and FocalLoss. Among them, we defined three learnable parameters as the weighted values of the three loss functions, with initial values of 0.3, 0.4, and 0.3, respectively. Subsequently, the loss module jointly participates in training and updates the weighted values in order to find the optimal value at a low cost. The effectiveness of this method has been validated in the literature [35].

(3) $$Loss=w_{1}\times CE\;Loss+w_{2}\times Dice\;Loss+w_{3}\times Focal\;Loss$$

## 3. Results

### 3.1. Datasets

#### 3.1.1. Sources

**Figure 3:**
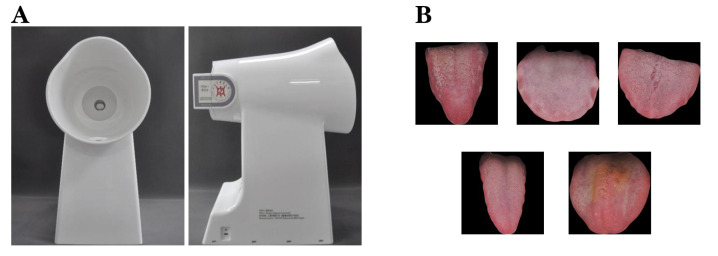
(**A**) TFDA-1 digital tongue diagnosis equipment. (**B**) Dataset examples.

In view of the urgent need for tongue image characterization technology, the National Key Research and Development Program of China “Research and Development of Intelligent Tongue Diagnosis System for Traditional Chinese Medicine” was created. This study used the TFDA-1 digital tongue diagnosis equipment independently developed by the project team [36] to obtain tongue images of 300 subjects at the Beijing University of Chinese Medicine, Dongzhimen Hospital. The major equipment includes a CCD camera (M mode, shutter speed 1/125 s, aperture value F6.3, ISO sensitivity 200, central focus metering, automatic white balance), an LED light source (color temperature 5000 K, color rendering index 97), a light hood, a stable base, and a curved reflector to capture tongue images with excellent quality and consistency. To ensure standardization and accuracy during data collection, all images were obtained by researchers who underwent strict standardization training. Collection procedures included the following: 1. Setting the shooting parameters and using 75% medical alcohol to fully disinfect the equipment; 2. Asking the subject to place their chin on the professional mandibular bracket of the tongue diagnostic instrument, remain relaxed, open their mouth, and stretch the tongue body, trying to completely flatten the tongue surface while at the same time slightly touching the center of the tongue surface to the camera screen to complete the image collection; 3. Carefully checking the captured images to ensure that the tongue surface is intact and free of tension and has no fogging or haziness and to prevent light leakage, overexposure, or underexposure. Images that do not meet the above standards need to be recaptured; 4. Using a tongue segmentation framework [37] to segment the tongue image from the face and obtain an image containing only the tongue body and tongue coating (Figure 3B) to construct a dataset.

All subjects participating in the study fully understood and signed the informed consent form and clearly understood the purpose and significance of this study.

#### 3.1.2. Labeling

The tongue coating labeling of 300 tongue images was completed and cross-verified by two attending doctors. The labels are divided into tongue coating and background. A resident physician performed final verification of inconsistent labeling. Tongue coating verification complies with the “China GB/T 20348-2006 National Standard” [38] and the “Differential Diagnosis of Symptoms of Traditional Chinese Medicine” [39].

### 3.2. Evaluation Metrics

We can consider the image segmentation problem as a pixel-by-pixel classification problem. For tongue images, each pixel belongs to either the tongue coating category or the background category. Therefore, we use true positive (TP) to represent the number of tongue coating pixels classified as tongue coating, false positive (FP) to represent the number of tongue coating pixels classified as background, true negative (TN) to represent the number of background pixels classified as tongue coating, and false negative (FN) to represent the number of background pixels classified as background. We use five metrics, i.e., accuracy (4), precision (5), dice (6), recall (7), and IoU (8) to test the tongue coating segmentation effect.

- Accuracy: This indicates that the model correctly predicts the number of pixels in proportion to all pixels.
- Precision: The ratio of the number of tongue coating pixels correctly predicted by the model to the actual number of tongue coating pixels.
- Recall: The ratio of the number of pixels correctly predicted by the model to the actual number of pixels in the tongue coating.
- Dice: The precision reflects the model’s ability to distinguish non-tongue coating area. The higher the precision, the stronger the model’s ability to distinguish non-tongue coating area. The recall reflects the model’s ability to recognize the tongue coating area. The higher the recall, the stronger the model’s ability to recognize the tongue coating area. Dice is the average sum of the two. The higher the dice, the more robust the model becomes.
- IoU: IoU is a commonly used evaluation metric used to calculate the ratio between the intersection and union of two sets of predicted segmentation results and true segmentation results.

(4) $$\begin{array}{cc} Accuracy & =\frac{TP+TN}{TP+TN+FP+FN} \end{array}$$

(5) $$\begin{array}{cc} Precision & =\frac{TP}{TP+FP} \end{array}$$

(6) $$\begin{array}{cc} Dice & =\frac{2\times TP}{2\times TP+FP+FN} \end{array}$$

(7) $$\begin{array}{cc} Recall & =\frac{TP}{TP+FN} \end{array}$$

(8) $$\begin{array}{cc} IoU & =\frac{TP}{TP+FP+FN} \end{array}$$

### 3.3. Implementation Details

Our model was implemented based on PyTorch and trained on the Ubuntu 22.04.2 LTS operating system, equipped with a 2.10 GHz Intel Xeon E5-2683 CPU, 320G RAM, and 24G video memory NVIDIA Quadro RTX6000 GPU. The Adam algorithm was used for optimizing a learning rate of 0.01, momentum of 0.9, weight decay of 1 $\times 10^{-4}$, and batch size of 6. The image input size was 224 × 224. Both transformer (ViT) and ResNet-50 were pretrained on ImageNet [20]. The model may have been undertrained due to the small size of the dataset. Therefore, we augmented the images by random rotation and image flipping. To fully use the limited data and test the generalization capacity of the model, the five-fold cross-validation method was used to divide the dataset. Unlike simply dividing into training and test sets, cross-validation can minimize the possibility that the results will be influenced by the specific way of dividing the data.

### 3.4. Comparative Study

Based on open-source code, we used Unet [19], UNet++ [26], and SegNet [40] to perform five-fold cross-validation on the same dataset and compared the results with our model. Table 1 shows the results. Our model achieved the best performance, significantly outperforming the representative UNet method. The accuracy, precision, dice, recall, and IoU increased by 3.04%, 4.14%, 2.56%, 0.48%, and 4.5%. Table 2 shows the standard deviation of each group of indicators after five-fold cross-validation. The standard deviation of our model is significantly better than that of the reference model, indicating its stable performance.

**Figure 4:**
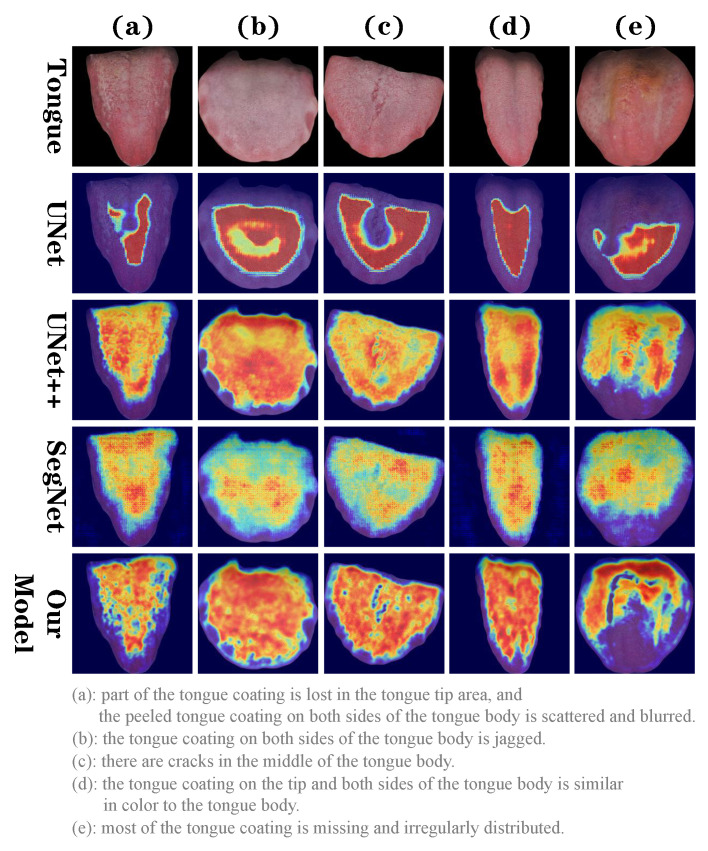
Attention heat map generated by extracting the weights of the last convolutional layer using GradCAM.

To intuitively compare the difference in tongue coating attention between our model and the reference model, GradCAM [41] was used to extract the weights of the last convolutional layer of each model to generate an attention heat map. As shown in Figure 4, the model pays more attention to the red part, which contributes more to the prediction results; it pays less attention to the yellow part; the blue part contributes less to the prediction results, and the model considers this area to be redundant information. We selected five representative tongue images from the dataset: (a) part of the tongue coating is lost in the tongue tip area, and the peeled tongue coating on both sides of the tongue body is scattered and blurred; (b) the tongue coating on both sides of the tongue body is jagged; (c) there are cracks in the middle of the tongue body; (d) the tongue coating on the tip and both sides of the tongue body is similar in color to the tongue body; (e) most of the tongue coating is missing and irregularly distributed. The heat map shows that UNet pays more attention to the parts of the tongue image with apparent tongue coating characteristics and is extremely concentrated; however, it pays insufficient attention to the edges and details with obvious errors. For example, in (e), the focus is on the missing part of the tongue coating; in (c), although the crack in the middle of the tongue coating is identified, the edge is rough, and the tongue coating near the crack is lost; and in (b), no attention is paid to the tooth marks on the edge of the tongue body. Unlike UNet, UNet++ and SegNet focus on the tongue coating, and their attention to edges and details is improved as well. For example, the tooth marks on the edge of the tongue body in (b) and the cracks in the middle of the tongue body in (c) are both clearer than with UNet; however, the attention to the mixed tongue coating in (a) remains unclear. Both are also wrong in focusing on the loss of tongue coating on the tip of the tongue in (e). The attention of our model to the tongue coating is more consistent with the actual situation, with a more precise and clear attention distribution. It shows that whereas the transformer extracts semantic information in high-level features of the encoder, SFP and VRE effectively reduce redundant information in low-level features and improve spatial detail information, allowing accurate focus on tongue coating features including peeling, tooth marks, and cracks.

**Figure 5:**
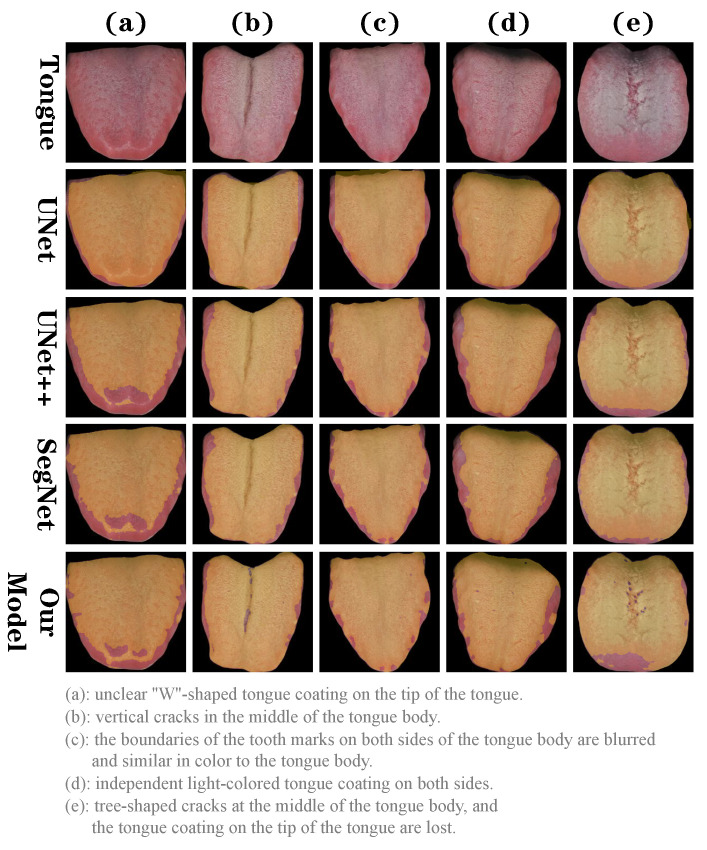
Visual segmentation results of tongue coating by models.

To visually assess and compare the performance of each model on the tongue coating segmentation task, we selected five more complex tongue images from outside the dataset: (a) images with unclear “W”-shaped tongue coating on the tip of the tongue; (b) images with vertical cracks in the middle of the tongue body; (c) the boundaries of the tooth marks on both sides of the tongue body are blurred and similar in color to the tongue body; (d) images with independent light-colored tongue coating on both sides; (e) images with tree-shaped cracks at the middle of the tongue body, and the tongue coating on the tip of the tongue are lost. As shown in Figure 5, UNet does not provide satisfactory segmentation results for the five tongue images. UNet++ and SegNet predicted the “W”-shaped tongue coating in (a); however, the details were not as precise as with our model and these models did not correctly predict the cracks in (b,e), and the tongue coating on both sides of the tongue body in (d). Our model provides satisfactory results; however, it also has some limitations. For instance, there is overfitting in predicting the tongue coating on both sides of the tongue. This is our future direction of improvement.

### 3.5. Ablation Study

The ablation study aims to investigate the efficacy of SFP and VRE in tongue coating segmentation. To this end, we conducted a series of experiments.

Using TransUNet as the baseline, we analyzed the contribution of SFP and VRE in improving model segmentation accuracy. Table 3 shows that unlike TransUNet, SFP improves accuracy, precision, dice, recall, and IoU by 0.47%, 0.48%, 0.3%, 0.17%, and 0.52%, respectively. Based on SFP, unlike TransUNet, the metrics of VRE increased by 0.58%, 0.75%, 0.45%, 0.16%, and 0.77%, respectively. The results indicate that SFP and VRE improve the tongue coating segmentation accuracy of the model.

Additionally, to reduce the number of parameters and calculations, we tried to alter the feature extraction level of ResNet-50 in the encoder from five levels to four levels. The results are shown in Table 4. The results of the five-level feature extraction network are inferior to those of the four-level feature extraction network, which may be attributed to the smaller feature resolution of the input transformer (7 × 7) and overfitting.

**Figure 6:**
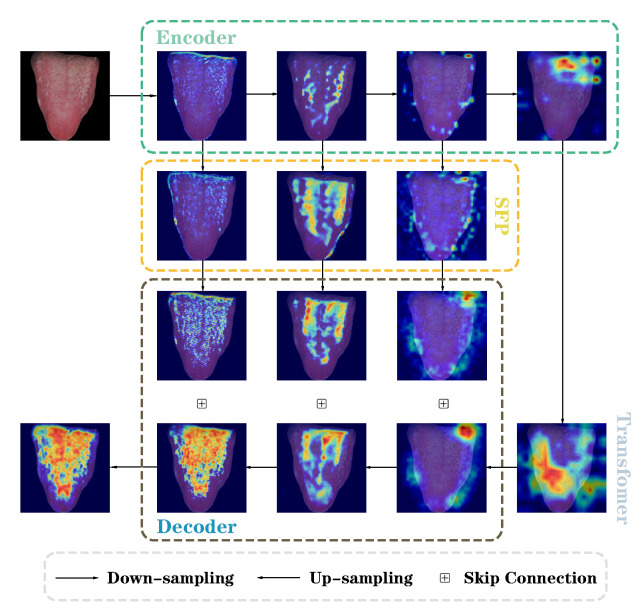
Attention heat map of each stage of the model generated by GradCAM.

Figure 6 shows the attention heat map at each stage of the model generated using GradCAM. The low-level features of the encoder expand the scope of attention after SFP and pay more attention to the edges of the tongue body. VRE improves the detailed information in the local space, allowing the model to focus on areas that are difficult to distinguish, including the edges of the tongue coating.

#### 3.5.1. SFP

Table 5 displays the results. We use TransUNet as the baseline and perform addition and subtraction operations, respectively, between the encoder features of each level and the level difference sum in SFP. All the metrics are better than addition operations when applying subtraction operations. SFP effectively reduces the redundant information in the low-level features of the encoder and pays more attention to the detailed information in the local space. Multiscale feature extraction is a common problem in the field of computer vision, and we believe that SFP can promote more research on subtraction operations in the future.

#### 3.5.2. VRE

The results are shown in Table 6. We investigated the effect of independently using LVC and VRE in series as well as parallel, respectively, on tongue coating segmentation accuracy in TransUNet using SFP. In the series structure, we inputted the encoder features of each level after SFP into two LVCs connected in series and obtained the features of each level with channel numbers 512, 256, and 64, respectively, participating in the decoder. In the parallel structure, we first inputted the encoder features of each level after SFP into two identical LVCs. Subsequently, the output features were fused and then inputted into an LVC. Eventually, the features of each level with channel numbers 512, 256, and 64 were obtained, respectively, and participated in the decoder. The findings indicate that the VRE of the series structure is slightly better than the parallel structure; besides, the number of parameters and computations is smaller. Additionally, connecting two LVCs in series is better than individually applying an LVC. Therefore, we constructed VRE in series to ensure segmentation accuracy and prevent overfitting.

## 4. Discussion

The main aim of this study was to embed SFP and VRE into TransUNet through design and experiments and conduct in-depth analysis and quantitative evaluation of their capacity to improve tongue coating segmentation tasks. Currently, several models have been developed for accurate tongue body segmentation [16,42,43,44]. However, to the best of our knowledge, few studies have investigated tongue coating segmentation models. As an important part of tongue appearance, the tongue coating influences the occurrence and prognosis of diseases [3,4,5,6]. Tongue coating segmentation is also a key step in intelligent tongue diagnosis. The color of the tongue coating is similar to that of the tongue body, and its boundaries are blurred, irregular in shape, and unevenly distributed, making the segmentation details of the tongue coating challenging. Tongue coating segmentation presents a significant challenge due to the complex interplay between features. To solve this problem, based on its advantages in the field of medical image segmentation, we use TransUNet as the basic architecture and utilize the advantages of a transformer to effectively capture the overall features of the tongue coating. To address the limitations of the transformer in capturing fine details, we introduce two complementary modules, spatial feature purification (SFP) and vital region enhancement (VRE), to reduce redundant information in low-level features and reduce significant differences between high-level and low-level features, making them more effectively fused. We pass the low-level features of the encoder to SFP and VRE for processing, while the high-level features are processed by the transformer. Finally, high-level features and low-level features are integrated through a U-shaped structure and jointly run in the decoder to generate tongue coating segmentation results. In recent years, this method of entrusting features to different modules or networks for processing and finally fusing them has become more common because it can not only effectively utilize feature information at different scales and improve the model’s understanding of complex scenes but also enhance the comprehensiveness and robustness of feature representation through complementary subnets. Lei Zhou et al. [45] designed a multibranch ensemble network consisting of two subnets for brain segmentation in MRI. Two subnets that exhibit complementary semantic clues are combined and designed for breast tumor segmentation. Finally, the hierarchical integration module can effectively integrate information from the two subnets. The results indicate that the proposed method has superior performance compared to state-of-the-art methods, especially in segmenting NME and small-sized tumors. Li Zihan et al. [46] designed a ScribFormer framework consisting of a three-branch network, which combines CNN, transformer, and attention-guided class activation map (ACAM) branches. ScribFormer utilizes the transformer branch to refine convolutional features and the ACAM generated by the CNN branch. It generates high-quality pixel-level segmentation results simply and efficiently. Comparative experimental results show that our model is better than U-Net, U-Net++, and SegNet in terms of accuracy, precision, dice, recall and IoU metrics. Moreover, after five-fold cross-validation, the standard deviation of each group of metrics is the smallest, indicating that its performance is stable. Both the attention heat map generated by GradCAM and the visual segmentation indicate show that our model can better cope with complex tongue phenomena such as tooth marks, cracks, peeling, and other irregular tongue coatings. These results validate the superior performance of our model in tongue coating segmentation, which may be attributed to its ability to enhance detail information in low-level features of the encoder by SFP and VRE, thereby capturing semantic information in high-level features without ignoring spatial information that plays an important role in fuzzy boundaries in tongue coating segmentation. The GradCAM heat map presented in Figure 6 also indicates that SFP and VRE expand the attention span and enhance the model’s attention to spatial detail information, which can cope with the challenge of complex tongue coating segmentation.

## 5. Conclusions

This paper proposes a tongue coating segmentation method leveraged on the TransUNet model, which allows accurate segmentation of complex tongue coatings such as tooth marks, cracks, and peelings. Tongue coating segmentation is a key component of intelligent tongue diagnosis. To address this challenge, we reexamined the feature processing methods at different levels in multiscale features. We inputted the first three levels of the four-level features of the encoder into SFP and VRE as low-level features to enhance the detailed information in the local space. The last level is used as a high-level feature inputted into the transformer to model the relationships between features through its self-attention mechanism, which is not limited by local interactions, thus fully utilizing contextual information and capturing long-distance dependencies. Comparative experimental results show that the segmentation effect of our model is better than U-Net, U-Net++, and SegNet on the same dataset. Ablation experimental tests also indicate that SFP and VRE improve the detail segmentation accuracy of tongue coating edges to a certain extent. Nevertheless, our study has some limitations. Firstly, the dataset used is small and the advantages of the transformer are not fully utilized. Finding out how to enhance the accuracy and robustness of the model while increasing the size of the dataset remains a challenging task. Secondly, generalization of the performance of the proposed model was not verified in other new segmentation environments, such as directly segmenting the tongue body and tongue coating from the human face. In future work, we aim to expand the scale of the dataset and enhance label accuracy while using data enhancement and data selection methods to improve the quality of the dataset. Thirdly, we shall continue to optimize the model performance to achieve higher segmentation accuracy on larger datasets while maintaining its robustness and reducing computational complexity. Finally, strategies to improve the generalization ability of the model in different scenarios need to be developed.

## Figures and Tables

**Table 1:** Metrics of our model after five-fold cross-validation with UNet, UNet++, and SegNet models on the same dataset.

	Accuracy	Precision	Dice	Recall	IoU
Our model	96.36%	96.26%	96.76%	97.43%	93.81%
UNet	93.32%	92.12%	94.20%	96.95%	89.31%
UNet++	95.73%	94.39%	95.90%	97.04%	92.36%
SegNet	95.50%	95.54%	95.95%	96.62%	92.35%

**Table 2:** Standard deviation of our model after five-fold cross-validation with UNet, UNet++, and SegNet models on the same dataset.

	Accuracy	Precision	Dice	Recall	IoU
Our model	0.003757	0.008130	0.002809	0.004388	0.004859
UNet	0.021674	0.043071	0.018303	0.016150	0.030058
UNet++	0.003167	0.010994	0.004059	0.005874	0.006905
SegNet	0.005874	0.017500	0.006594	0.007607	0.010757

**Table 3:** Ablation experiment results.

	Accuracy	Precision	Dice	Recall	IoU
TransUNet	95.78%	95.51%	96.31%	97.27%	93.04%
+SFP	96.25%	95.99%	96.61%	97.44%	93.56%
+VRE	96.36%	96.26%	96.76%	97.43%	93.81%

**Table 4:** Impact of ResNet-50 with five layers and four layers on metrics.

	Accuracy	Precision	Dice	Recall	IoU
ResNet-50 (5 levels)	96.32%	96.17%	96.70%	97.41%	93.71%
ResNet-50 (4 levels)	96.36%	96.26%	96.76%	97.43%	93.81%

**Table 5:** Results when SFP adopts addition and subtraction operations, respectively.

	Accuracy	Precision	Dice	Recall	IoU
TransUNet	95.78%	95.51%	96.31%	97.27%	93.04%
+SFP(⊕)	96.23%	96.49%	96.60%	96.91%	93.54%
+SFP(⊖)	96.25%	95.99%	96.61%	97.44%	93.56%

**Table 6:** Results when VRE is constructed in series and parallel.

	Accuracy	Precision	Dice	Recall	IoU
TransUNet+SFP	96.25%	95.99%	96.61%	97.44%	93.56%
+LVC	96.33%	96.47%	96.70%	97.10%	93.71%
+VRE (parallel)	96.36%	96.21%	96.74%	97.45%	93.79%
+VRE (series)	96.36%	96.26%	96.76%	97.43%	93.81%

## Data Availability

Data underlying the results presented in this paper are not publicly available at this time but may be obtained from the authors upon reasonable request.
